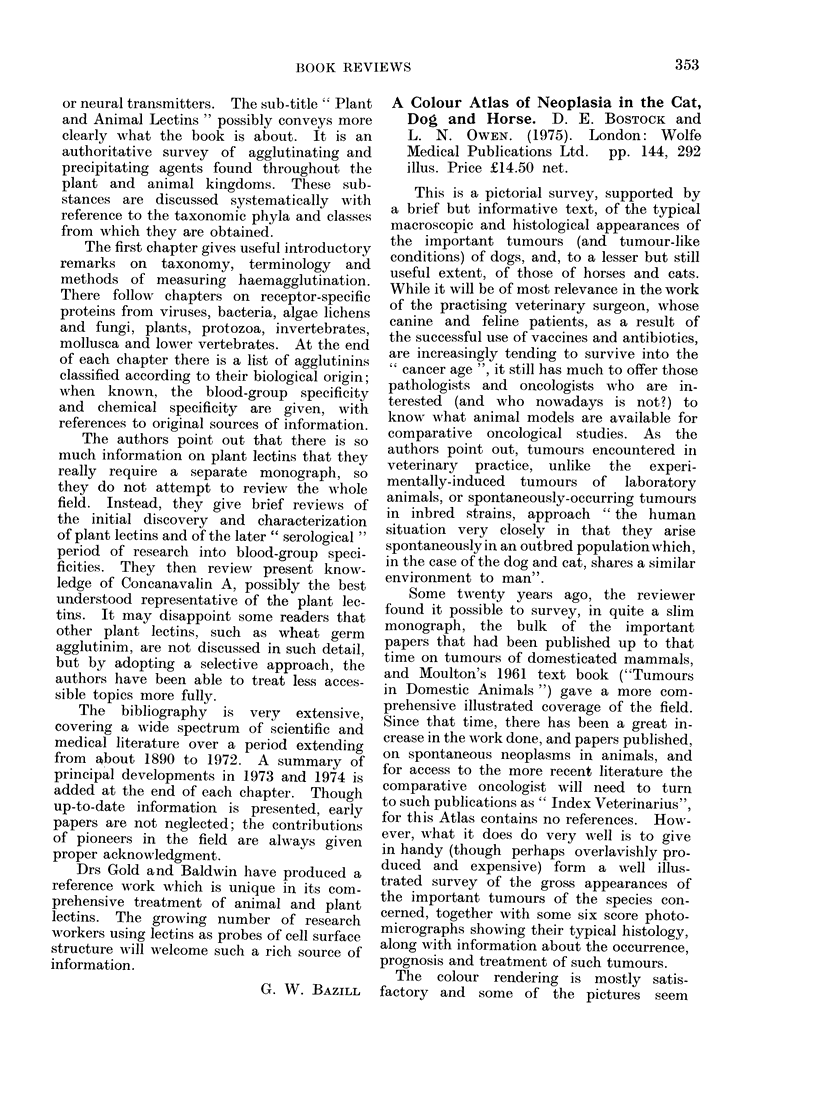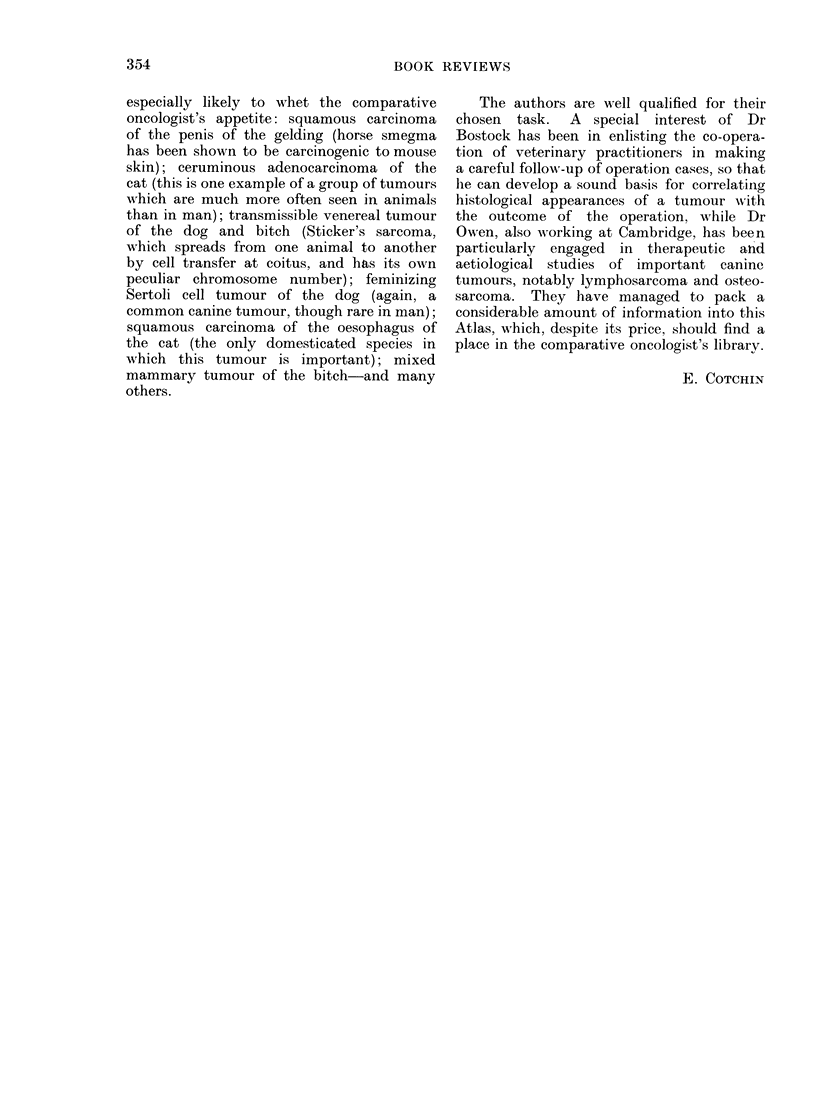# A Colour Atlas of Neoplasia in the Cat, Dog and Horse

**Published:** 1976-03

**Authors:** E. Cotchin


**A Colour Atlas of Neoplasia in the Cat, Dog and Horse.** D. E. Bostock and L. N. Owen. (1975). London: Wolfe Medical Publications Ltd. pp. 144, 292 illus. Price £14.50 net.

This is a pictorial survey, supported by a brief but informative text, of the typical macroscopic and histological appearances of the important tumours (and tumour-like conditions) of dogs, and, to a lesser but still useful extent, of those of horses and cats. While it will be of most relevance in the work of the practising veterinary surgeon, whose canine and feline patients, as a result of the successful use of vaccines and antibiotics, are increasingly tending to survive into the " cancer age ", it still has much to offer those pathologists and oncologists who are interested (and who nowadays is not?) to know what animal models are available for comparative oncological studies. As the authors point out, tumours encountered in veterinary practice, unlike the experimentally-induced tumours of laboratory animals, or spontaneously-occurring tumours in inbred strains, approach " the human situation very closely in that they arise spontaneously in an outbred population which, in the case of the dog and cat, shares a similar environment to man".

Some twenty years ago, the reviewer found it possible to survey, in quite a slim monograph, the bulk of the important papers that had been published up to that time on tumours of domesticated mammals, and Moulton's 1961 text book ("Tumours in Domestic Animals ") gave a more comprehensive illustrated coverage of the field. Since that time, there has been a great increase in the work done, and papers published, on spontaneous neoplasms in animals, and for access to the more recent literature the comparative oncologist will need to turn to such publications as " Index Veterinarius", for this Atlas contains no references. However, what it does do very well is to give in handy (though perhaps overlavishly produced and expensive) form a well illustrated survey of the gross appearances of the important tumours of the species concerned, together with some six score photomicrographs showing their typical histology, along with information about the occurrence, prognosis and treatment of such tumours.

The colour rendering is mostly satisfactory and some of the pictures seem especially likely to whet the comparative oncologist's appetite: squamous carcinoma of the penis of the gelding (horse smegma has been shown to be carcinogenic to mouse skin); ceruminous adenocarcinoma of the cat (this is one example of a group of tumours which are much more often seen in animals than in man); transmissible venereal tumour of the dog and bitch (Sticker's sarcoma, which spreads from one animal to another by cell transfer at coitus, and has its own peculiar chromosome number); feminizing Sertoli cell tumour of the dog (again, a common canine tumour, though rare in man); squamous carcinoma of the oesophagus of the cat (the only domesticated species in which this tumour is important); mixed mammary tumour of the bitch—and many others.

The authors are well qualified for their chosen task. A special interest of Dr Bostock has been in enlisting the co-operation of veterinary practitioners in making a careful follow-up of operation cases, so that he can develop a sound basis for correlating histological appearances of a tumour with the outcome of the operation, while Dr Owen, also working at Cambridge, has been particularly engaged in therapeutic and aetiological studies of important canine tumours, notably lymphosarcoma and osteosarcoma. They have managed to pack a considerable amount of information into this Atlas, which, despite its price, should find a place in the comparative oncologist's library.

E. Cotchin